# Supplementary material for: DeepMicro: deep representation learning for disease prediction based on microbiome data
Source: Sci Rep. 2020 Apr 7;10:6026. doi: 10.1038/s41598-020-63159-5 (PMC7138789; doi:10.1038/s41598-020-63159-5)
Supplement: Supplementary file 1 — Supplementary Information. [file 41598_2020_63159_MOESM1_ESM.pdf]

Supplementary Materials

DeepMicro: deep representation learning for disease prediction based on microbiome data

Min Oh[1] and Liqing Zhang[1,*]

[1]Department of Computer Science, Virginia Tech, Blacksburg, VA, USA

[*]Correspondence: lqzhang@cs.vt.edu

Contents

- **Figure S1.** Performance evaluation scheme
- **Figure S2.** Disease prediction performance for abundance profile-based models
- **Figure S3.** Disease prediction performance for different autoencoders based on abundance profile (assessed with AUC)
- **Figure S4.** Disease prediction performance of multi-layer perceptron without representation learning based on marker profile
- **Figure S5.** Disease prediction performance of multi-layer perceptron without representation learning based on abundance profile
- **Figure S6.** Impact of introducing negative samples into the training set on AUC
- **Figure S7.** Prediction performance changes over the increasing data points in the training set
- **Figure S8.** Disease prediction performance for marker profile-based models (fixed scale)
- **Table S1.** The best representation learning model structures for each dataset
- **Table S2.** Hyper-parameters used in grid search
- **Table S3.** Performance evaluation with area under precision-recall curve for IBD dataset

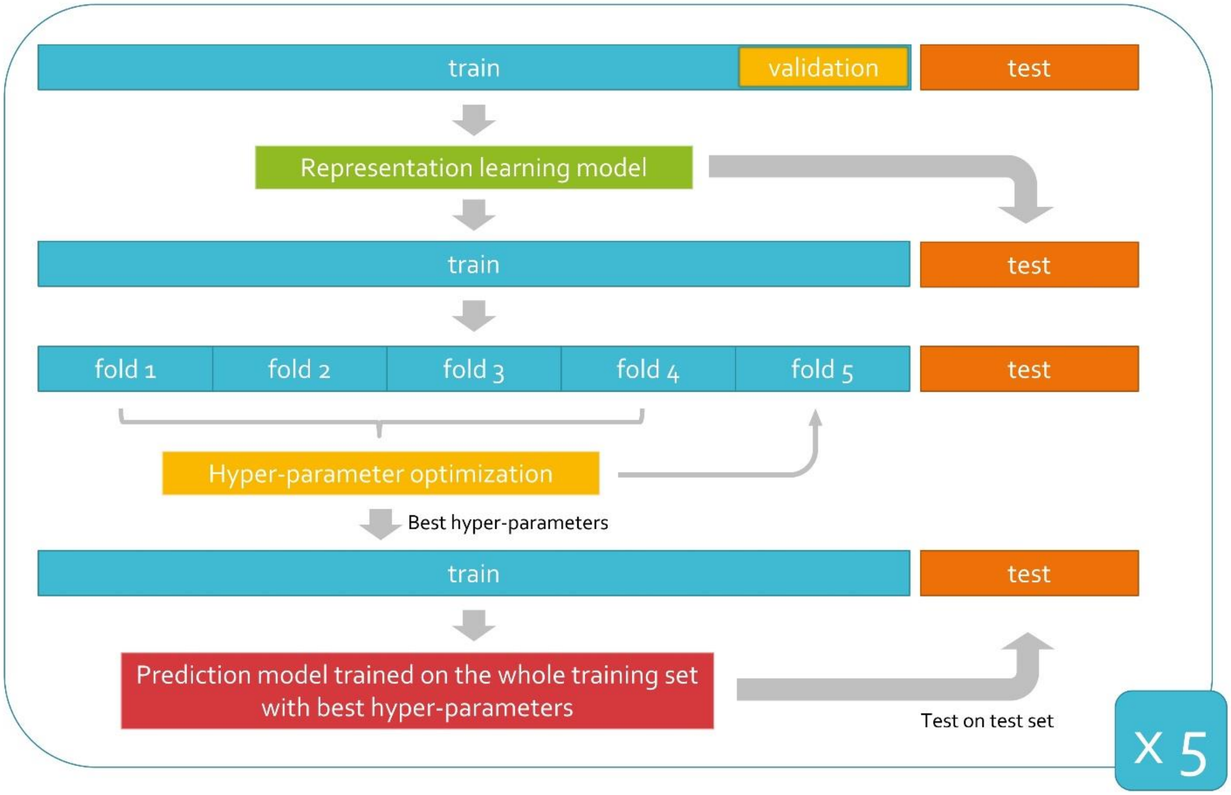

Figure S1. Performance evaluation scheme

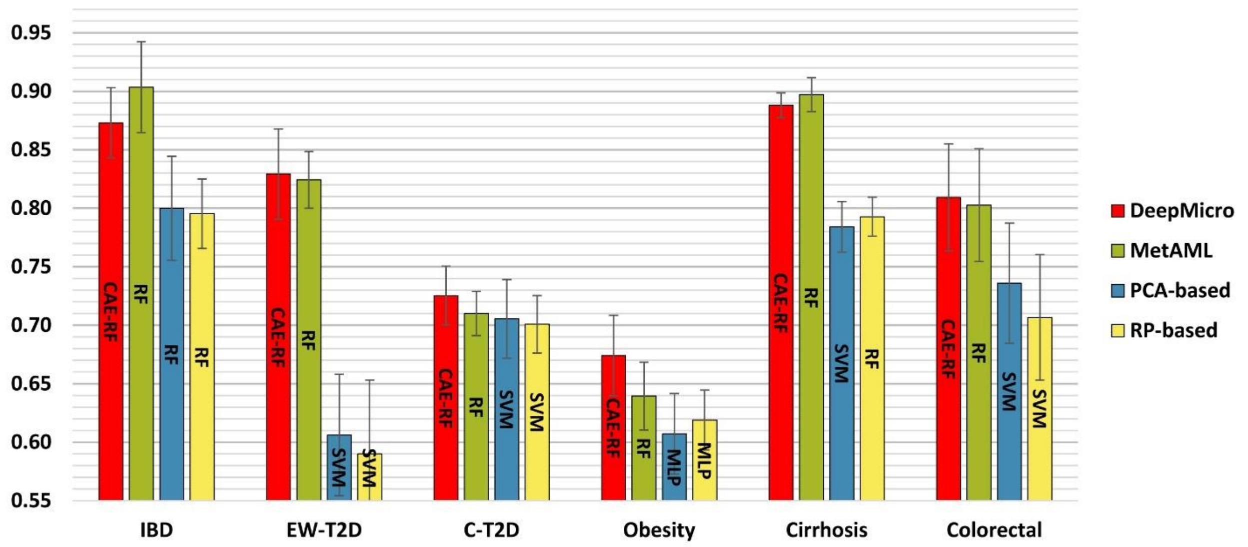

Figure S2. Disease prediction performance for abundance profile-based models. Prediction performance of various methods built on marker profile has been assessed with AUC. MetAML utilizes support vector machine (SVM) and random forest (RF), and the superior model is presented (green). Principal component analysis (PCA; blue) and gaussian random projection (RP; yellow) have been applied to reduce dimensions of datasets before classification. DeepMicro (red) applies shallow autoencoder (SAE), deep autoencoder (DAE), variational autoencoder (VAE), and convolutional autoencoder (CAE) for dimensionality reduction. Then SVM, RF, and multi-layer perceptron (MLP) classification algorithms have been used.

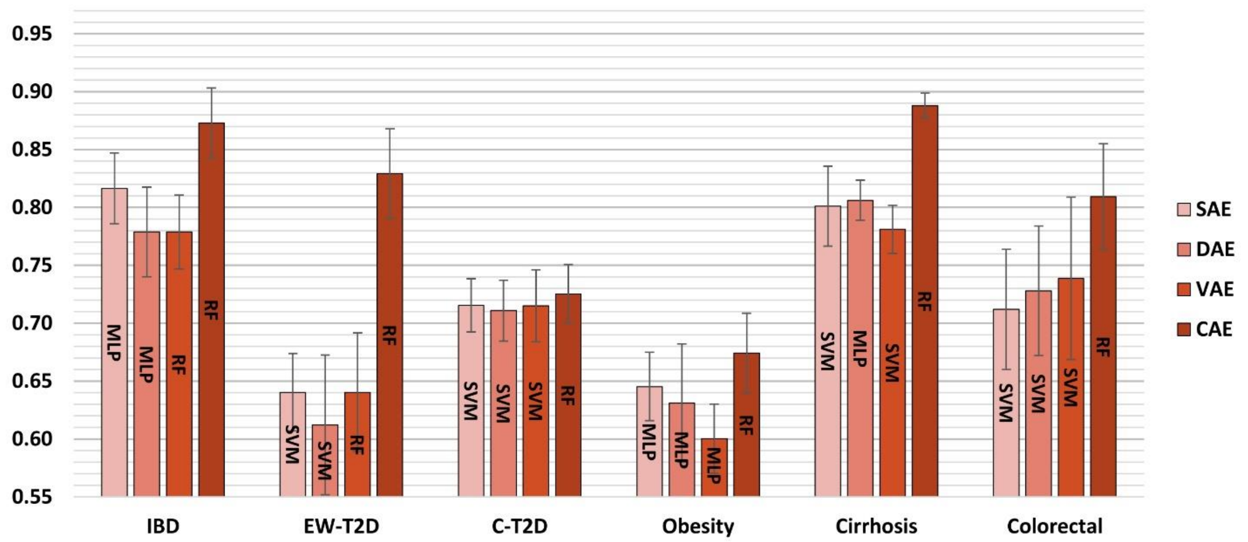

Figure S3. Disease prediction performance for different autoencoders based on abundance profile (assessed with AUC). Classifiers used: support vector machine (SVM), random forest (RF), and multi-layer perceptron (MLP); Autoencoders used: shallow autoencoder (SAE), deep autoencoder (DAE), variational autoencoder (VAE), and convolutional autoencoder (CAE)

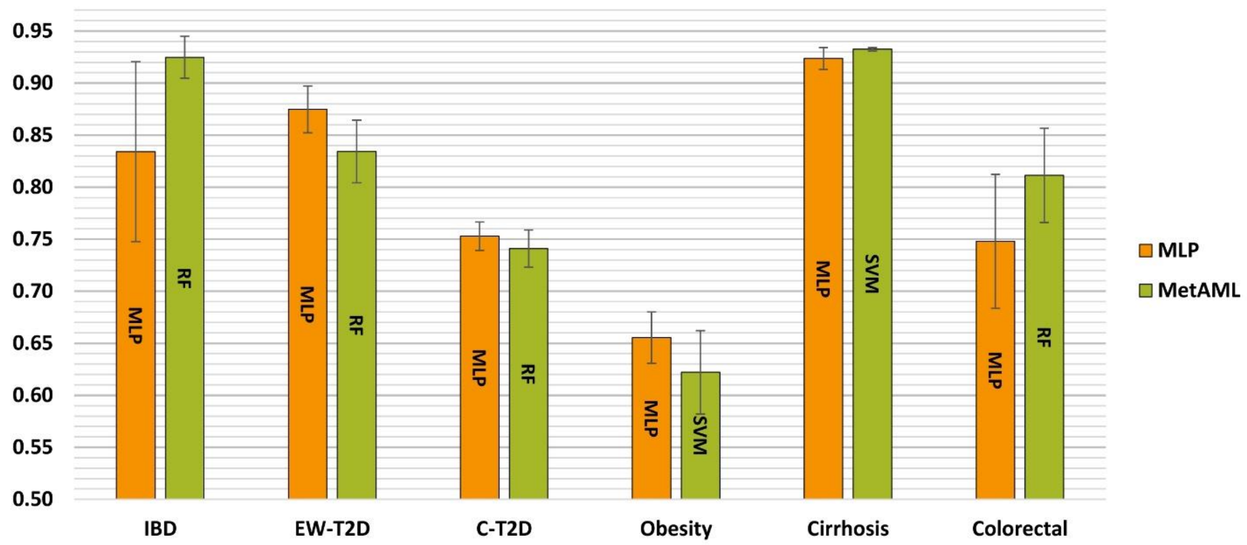

Figure S4. Disease prediction performance of multi-layer perceptron without representation learning based on marker profile

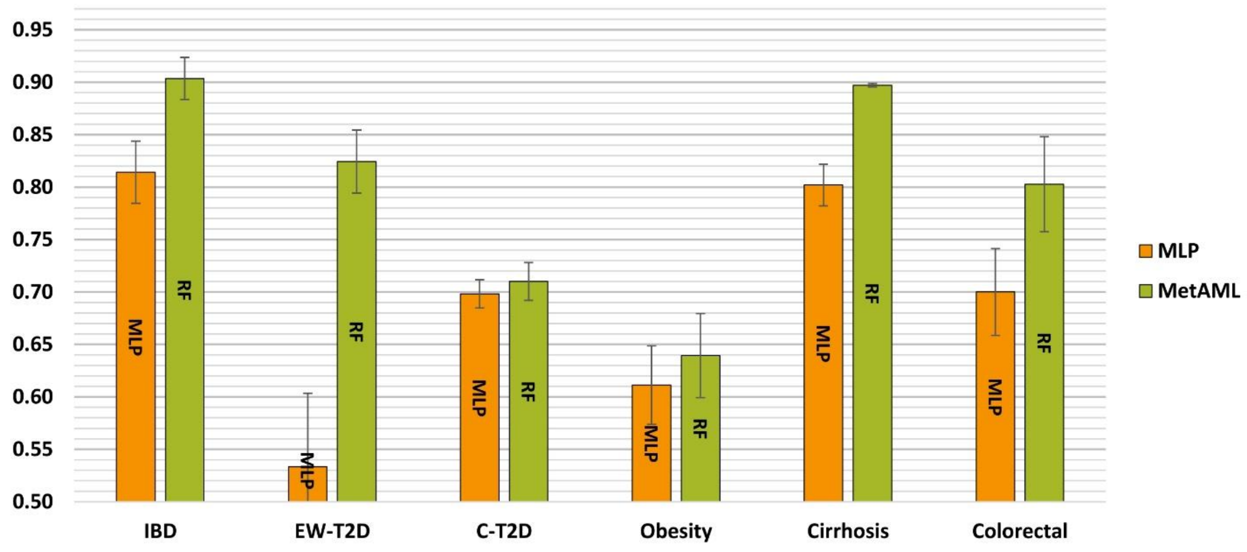

Figure S5. Disease prediction performance of multi-layer perceptron without representation learning based on abundance profile

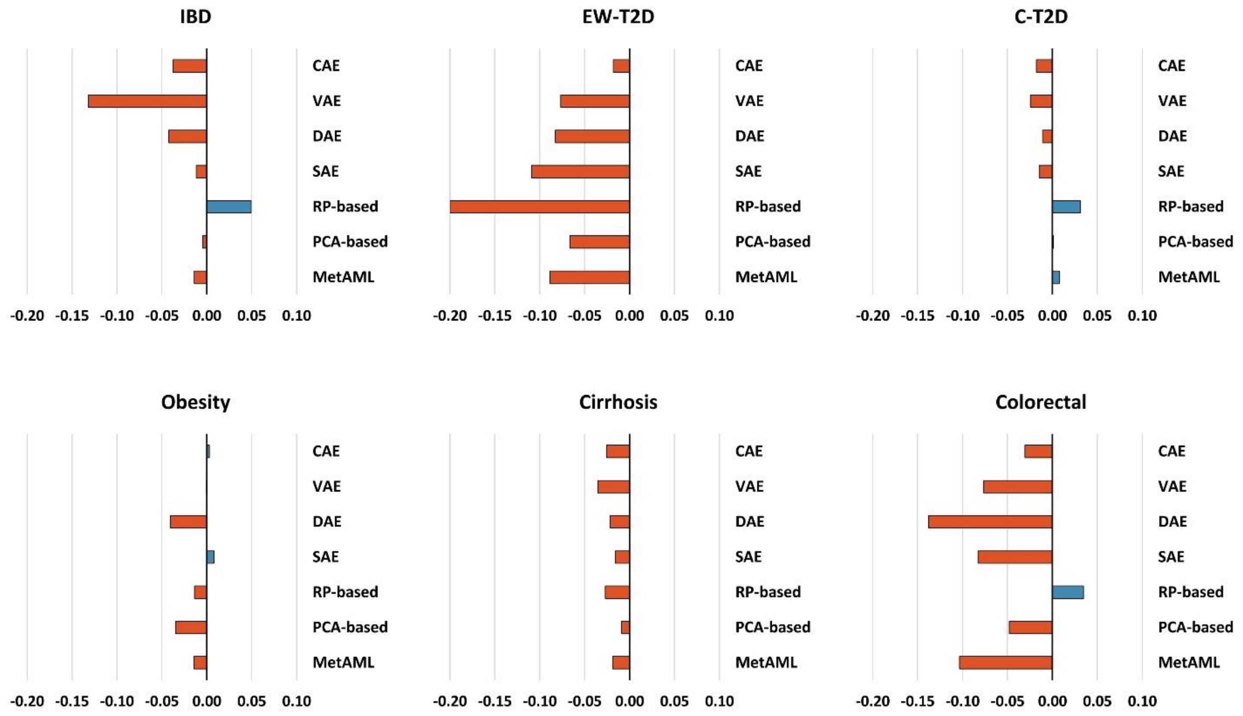

Figure S6. Impact of introducing negative samples into the training set on AUC

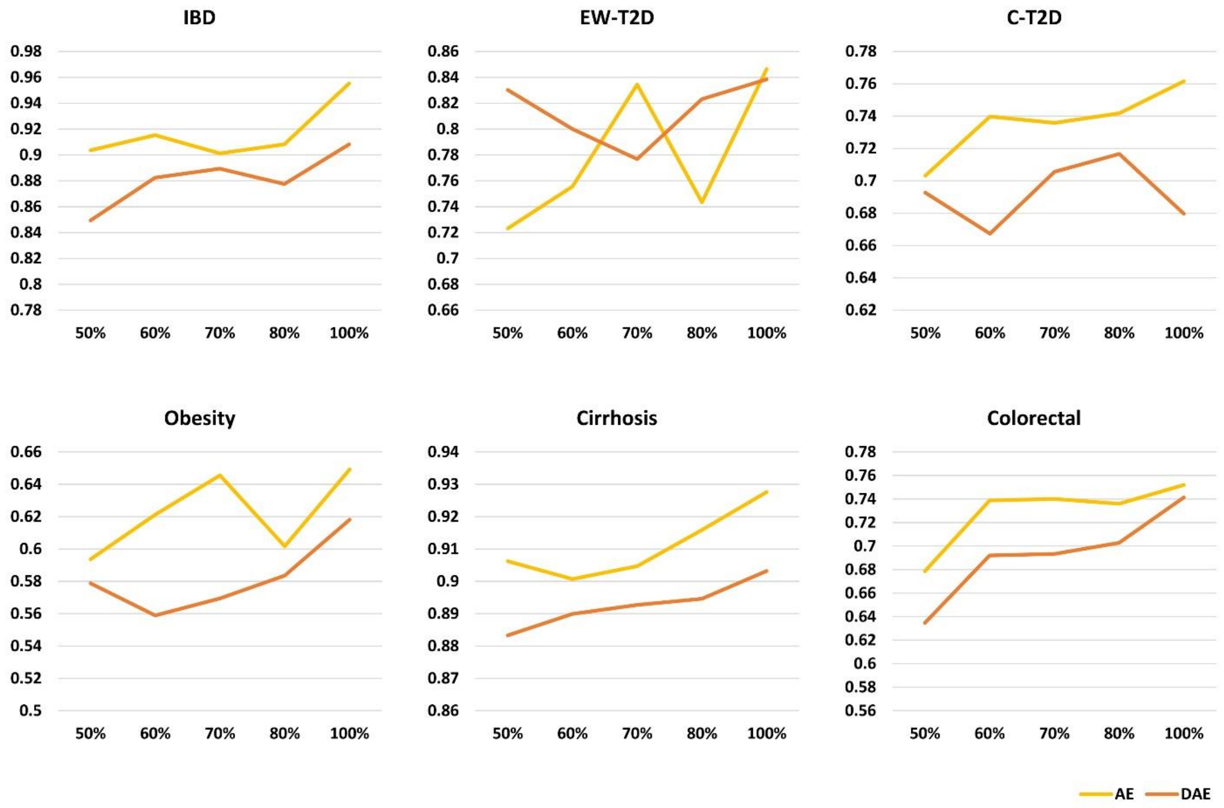

Figure S7. Prediction performance changes over the increasing data points in the training set

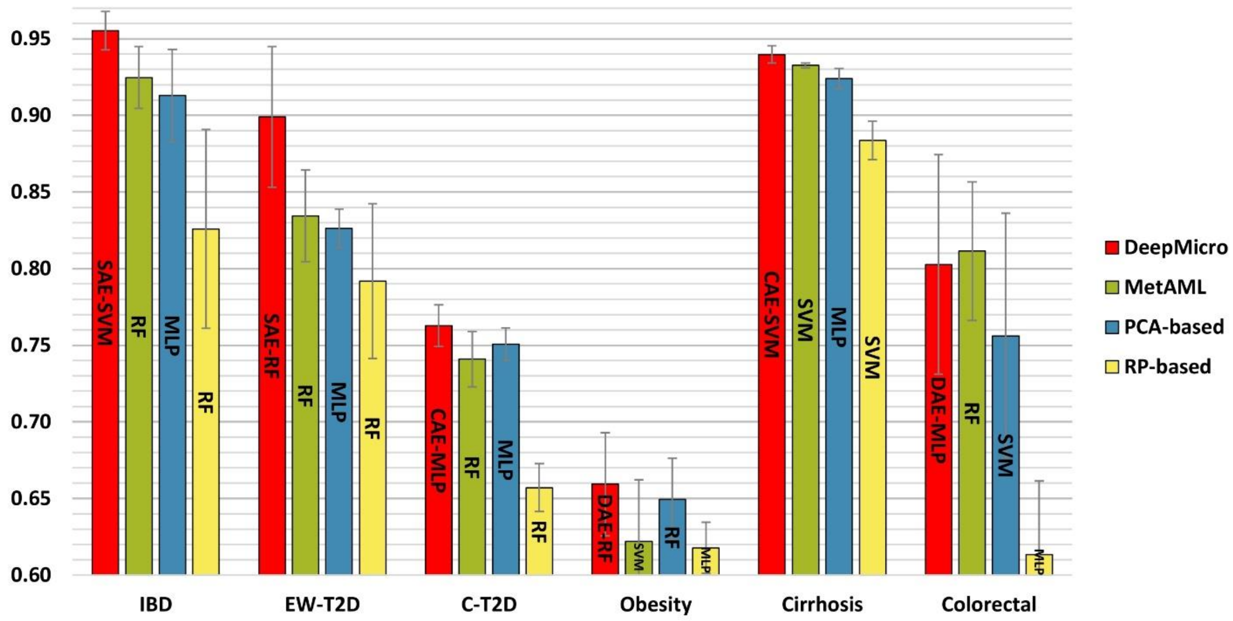

Figure S8. Disease prediction performance for marker profile-based models (fixed scale).

Table S1. The best representation learning model structures for each dataset

Microbiome Profile Type	Dataset	Size of Original Dim[#]	Representation Learning Model	Encoder Structure[*]	Size of Latent Dim	Classifier	Averaged AUC (Standard Error)	Averaged Accuracy (Standard Error)[**]
Strain-level marker profile	IBD	91,756	SAE	64	64	SVM	**0.955 (0.013)**	0.773 (0.000)
			DAE	512-256-128	128	RF	0.911 (0.046)	0.855 (0.027)
			VAE	128-4	4	MLP	0.899 (0.039)	0.818 (0.014)
			CAE	8-4	1,936	RF	0.929 (0.010)	0.882 (0.011)
	EW-T2D	83,456	SAE	256	256	RF	**0.899 (0.046)**	0.800 (0.047)
			DAE	256-128-64	64	RF	0.840 (0.029)	0.730 (0.041)
			VAE	256-16	16	SVM	0.853 (0.041)	0.600 (0.039)
			CAE	8-4	1,764	SVM	0.796 (0.014)	0.670 (0.030)
	C-T2D	119,792	SAE	512	512	SVM	0.762 (0.008)	0.664 (0.021)
			DAE	256-128	128	RF	0.702 (0.029)	0.649 (0.019)
			VAE	128-16	16	SVM	0.719 (0.019)	0.664 (0.022)
			CAE	4-2	968	MLP	**0.763 (0.014)**	0.710 (0.008)
	Obesity	99,568	SAE	512	512	MLP	0.658 (0.045)	0.624 (0.027)
			DAE	256-128	128	RF	**0.659 (0.034)**	0.635 (0.012)
			VAE	512-8	8	RF	0.599 (0.014)	0.639 (0.013)
			CAE	64-32	16,928	RF	0.622 (0.012)	0.655 (0.008)
	Cirrhosis	120,553	SAE	256	256	SVM	0.928 (0.006)	0.821 (0.020)
			DAE	512-256-128	128	SVM	0.903 (0.011)	0.809 (0.012)
			VAE	256-8	8	SVM	0.891 (0.016)	0.792 (0.029)
			CAE	16-8	3,872	SVM	**0.940 (0.006)**	0.864 (0.008)
	Colorectal	108,034	SAE	32	32	MLP	0.799 (0.058)	0.752 (0.039)
			DAE	512-256-128	128	MLP	**0.803 (0.072)**	0.728 (0.046)
			VAE	256-8	8	RF	0.737 (0.068)	0.696 (0.037)
			CAE	4-2-1	441	MLP	0.789 (0.044)	0.744 (0.033)
Species-level relative abundance profile	IBD	443	SAE	512	512	MLP	0.817 (0.031)	0.782 (0.017)
			DAE	512-256	256	MLP	0.779 (0.039)	0.791 (0.037)
			VAE	32-8	8	RF	0.779 (0.032)	0.782 (0.017)
			CAE	32-16-8	3,872	RF	**0.873 (0.030)**	0.809 (0.017)
	EW-T2D	381	SAE	256	256	SVM	0.640 (0.033)	0.630 (0.037)
			DAE	1024-512	512	SVM	0.612 (0.060)	0.580 (0.026)
			VAE	64-8	8	RF	0.640 (0.051)	0.570 (0.047)
			CAE	16-8	3,200	RF	**0.829 (0.039)**	0.740 (0.037)
	C-T2D	572	SAE	64	64	SVM	0.715 (0.023)	0.635 (0.030)
			DAE	128-64	64	SVM	0.711 (0.026)	0.649 (0.026)
			VAE	512-16	16	SVM	0.715 (0.031)	0.652 (0.031)
			CAE	4-2-1	576	RF	**0.725 (0.025)**	0.644 (0.025)
	Obesity	465	SAE	128	128	MLP	0.645 (0.030)	0.659 (0.017)
			DAE	1024-512	512	MLP	0.631 (0.051)	0.612 (0.020)
			VAE	256-4	4	MLP	0.600 (0.030)	0.635 (0.012)
			CAE	4-2	968	RF	**0.674 (0.034)**	0.655 (0.013)
	Cirrhosis	542	SAE	32	32	SVM	0.801 (0.035)	0.723 (0.050)
			DAE	1024-512	512	MLP	0.806 (0.017)	0.706 (0.030)
			VAE	512-8	8	SVM	0.781 (0.021)	0.711 (0.035)
			CAE	16-8-4	1,461	RF	**0.888 (0.011)**	0.830 (0.029)
	Colorectal	503	SAE	256	256	SVM	0.712 (0.052)	0.672 (0.037)
			DAE	256-128	128	SVM	0.728 (0.056)	0.648 (0.046)
			VAE	512-8	8	SVM	0.739 (0.070)	0.632 (0.037)
			CAE	8-4	2,116	RF	**0.809 (0.046)**	0.704 (0.020)

[#]Dim: Dimension; SAE: Sallow Autoencoder; DAE: Deep Autoencoder; VAE: Variational autoencoder; CAE: Convolutional autoencoder; SVM: Support Vector Machine; RF: Random Forest; MLP: Multi-layer Perceptron

[*]The number of units for SAE, DAE, and VAE; The number of filters for CAE; Layers are separated by a delimiter "-"

[**]Note that as the models are optimized for AUC performance, not accuracy, it is required to re-train our models by optimizing accuracy if you need to directly compare the accuracy performance with your models.

Table S2. Hyper-parameters used in grid search

Purpose	Method	Hyper-parameter tuned with grid search	Used values
Learning Representation	SAE	Size of latent layer	32, 64, 128, 256, 512
	DAE	Size of latent layer	32, 64, 128, 256, 512
		# of hidden layers in both encoder and decoder	1, 2
	VAE	Size of latent layer	4, 8, 16
		# of hidden units in the hidden layers	32, 64, 128, 256, 512
	CAE	# of convolutional layers	2, 3
		# of filters in the first conv layer	4, 8, 16, 32, 64
Learning Classifier	SVM	Penalty parameter C	2^{-5}, 2^{-3}, 2^{-1}, 2, 2^{3}, 2^{5}
		RBF kernel coefficient	2^{-15}, 2^{-13}, 2^{-11}, 2^{-9}, 2^{-7}, 2^{-5}, 2^{-3}, 2^{-1}, 2, 2^{3}
	RF	# of trees (estimators)	100, 300, 500, 700, 900
		The minimum number of samples in a leaf node	1, 2, 3, 4, 5
		Split criteria	Gini impurity, information gain
	MLP	# of hidden layers	1, 2, 3
		# of hidden units in the first layer	10, 30, 50, 100
		Dropout rate	0.1, 0.3
		# of epochs	30, 50, 100, 200, 300

[#] SAE: Sallow Autoencoder; DAE: Deep Autoencoder; VAE: Variational autoencoder; CAE: Convolutional autoencoder; SVM: Support Vector Machine; RF: Random Forest; MLP: Multi-layer Perceptron

Table S3. Performance evaluation with area under precision-recall curve for IBD dataset

Microbiome profile type	Methods	Representation Learning	Classifier	AUC* (Standard Error)	AUPRC** (Standard Error)
Strain-level marker profile	DeepMicro	SAE	SVM	**0.9553** (0.013)	**0.8653** (0.035)
	MetAML	.	RF	0.8918 (0.033)	0.6770 (0.102)
	PCA-based	PCA	MLP	0.9223 (0.024)	0.7965 (0.059)
	RP-based	RP	RF	0.7882 (0.044)	0.5461 (0.079)
Species-level abundance profile	DeepMicro	CAE	RF	0.8659 (0.033)	0.7020 (0.064)
	MetAML	.	RF	0.9153 (0.037)	0.7915 (0.076)
	PCA-based	PCA	RF	0.8247 (0.034)	0.6220 (0.021)
	RP-based	RP	RF	0.7365 (0.052)	0.4980 (0.075)

[#] SAE: Sallow Autoencoder; CAE: Convolutional Autoencoder; PCA: Principal Component analysis; RP: Random Projection; SVM: Support Vector Machine; RF: Random Forest; MLP: Multi-layer Perceptron

*AUC: Area Under the receiver operating characteristic (ROC) Curve

**AUPRC: Area Under the Precision-Recall Curve
